# Interplay Between Mitochondrial Oxidative Disorders and Proteostasis in Alzheimer’s Disease

**DOI:** 10.3389/fnins.2019.01444

**Published:** 2020-01-29

**Authors:** Emilio Llanos-González, Ángel Andres Henares-Chavarino, Cristina María Pedrero-Prieto, Sonia García-Carpintero, Javier Frontiñán-Rubio, Francisco Javier Sancho-Bielsa, Francisco Javier Alcain, Juan Ramón Peinado, Yoana Rabanal-Ruíz, Mario Durán-Prado

**Affiliations:** ^1^Department of Medical Sciences, Faculty of Medicine, University of Castilla-La Mancha, Ciudad Real, Spain; ^2^Oxidative Stress and Neurodegeneration Group, Regional Centre for Biomedical Research, University of Castilla-La Mancha, Ciudad Real, Spain; ^3^Faculty of Medicine, University of Castilla-La Mancha, Ciudad Real, Spain

**Keywords:** Alzheimer’s disease, autophagy, endoplasmic reticulum stress, oxidative stress, proteasomal degradation, unfolded protein response

## Abstract

Although the basis of Alzheimer’s disease (AD) etiology remains unknown, oxidative stress (OS) has been recognized as a prodromal factor associated to its progression. OS refers to an imbalance between oxidant and antioxidant systems, which usually consist in an overproduction of reactive oxygen species (ROS) and reactive nitrogen species (RNS) which overwhelms the intrinsic antioxidant defenses. Due to this increased production of ROS and RNS, several biological functions such as glucose metabolism or synaptic activity are impaired. In AD, growing evidence links the ROS-mediated damages with molecular targets including mitochondrial dynamics and function, protein quality control system, and autophagic pathways, affecting the proteostasis balance. In this scenario, OS should be considered as not only a major feature in the pathophysiology of AD but also a potential target to combat the progression of the disease. In this review, we will discuss the role of OS in mitochondrial dysfunction, protein quality control systems, and autophagy associated to AD and suggest innovative therapeutic strategies based on a better understanding of the role of OS and proteostasis.

## Introduction

Alzheimer’s disease (AD) is a progressive neurodegenerative disorder characterized by the accumulation of senile plaques (SPs) and neurofibrillary tangles (NFTs) in several brain areas such as the hippocampus and frontal and parietal cortex, among others (Butterfield and Halliwell, 2019). This accumulation of abnormal aggregates formed by fibrillary amyloid-β (Aβ) peptide and phosphorylated tau proteins, respectively, leads to cognitive, behavioral, and functional impairment (Nelson et al., 2009). Clinically, AD is characterized by a progressive cognitive decline due to a notable loss of synapses and high rates of neuronal death (Nelson et al., 2009).

Despite increasing studies about the etiology and pathogenesis of AD, the question about the origin of the disease remains open. Multiple factors are considered to contribute to the onset and progression of the disease, but oxidative damage has gained importance in etiology fields (Cheignon et al., 2018). Indeed, oxidation–reduction equilibria or redox homeostasis is a key component in all biological processes, from bioenergetics to other essential functions such as vesicle transport or post-transcriptional modulations (Franco and Vargas, 2018). However, pronounced deviations, mainly toward oxidation, may cause damage to biomolecules, disrupting the physiological redox cell signaling and leading to a transient state known as oxidative stress (OS) (Halliwell and Gutteridge, 2015). Generally, OS is defined as an acute imbalance between the production of reactive oxygen species (ROS) and reactive nitrogen species (RNS) and antioxidant defense systems, enzymatic, and non-enzymatic (Halliwell and Gutteridge, 2015). ROS production is the result of cellular metabolism and environmental factors (i.e., cigarette smoke or pollutants) and is proportional to the cellular metabolic state (Uttara et al., 2009). By the way, the brain, which is a metabolically very active organ, has a low capacity for cell regeneration and is thus highly vulnerable to the harmful effects of the overproduction of ROS (Gilgun-Sherki et al., 2001; Uttara et al., 2009).

There is increasing literature supporting a principal role for mitochondrial dysfunction and oxidative damage in the pathogenesis of AD (Kausar et al., 2018). In many cases, oxidative damage promotes an upregulation of genes relating to mitochondrial metabolism, and ROS generated produces damages in mitochondrial DNA (mtDNA) and other mitochondrial components, inducing a dysfunctional mitochondrial state (Bhat et al., 2015). Other molecular pathways such as protein synthesis and folding in endoplasmic reticulum (ER) (Eletto et al., 2014) or autophagy (Peña-Oyarzun et al., 2018) are also coupled to the variations of redox homeostasis, leading in many cases to pathological responses.

In this review, we discuss the latest discoveries highlighting the role of OS in AD and its impact in mitochondrial dysfunction, proteostasis, and autophagic response.

## Oxidative Stress and Mitochondrial Dysfunction in Alzheimer’s Disease

Aging, genetic, and some environmental factors could imbalance the oxidative-redox system to a pro-oxidant state, increasing the ROS and RNS levels (Gomes et al., 2018). Mitochondria is the main consumer of oxygen, and hence, one of the main sources of ROS at the intracellular level, producing between 1% and 5% of total cellular ROS in normal, physiological, conditions (Wei et al., 2001). Although, an overproduction of free radicals such as superoxide anion (O_2_^–^) and hydroxyl radical (OH) can generate unrepaired damage to mtDNA (Han and Chen, 2013), many studies pointed out the regulation of nuclear components through ROS signaling (Larosa and Remacle, 2018). For example, mitochondrial ROS can upregulate the expression of assembly factors of I, II, III, and IV complexes (Carden et al., 2017). In this way, mitochondrial ROS communicate with the nucleus in a bidirectional fashion.

Under stress conditions, metabolic challenges, genomic instability, and cellular damages could be observed linked to an overproduction of ROS (Shimura and Kunugita, 2016). For instance, in AD where OS is considered a key step in the disease progression, ROS increase the expression of β-secretase through activation of p38 mitogen-activated protein kinase (MAPK) 23 (Giraldo et al., 2014) and increase abnormal tau phosphorylation by activation of glycogen synthase kinase 3, GSK3 (Xiong et al., 2013). Redox proteomics of AD brain tissue revealed oxidative modification in enzymes involved in glucose metabolism, which often leads to a decrease in their activity (Sultana et al., 2007). In addition, oxidative modifications to creatine kinase, an enzyme that maintains ATP levels in neurons, and ATP synthase in brain mitochondria help to explain the relationship between ROS overproduction and metabolism in mild cognitive impairment (MCI) and AD (Sultana et al., 2007). The consequences of this oxidative modification–induced decrease on ATP production in AD are spotted in the neuron’s ability to maintain biological functions such as synapse assembly, generation of action potentials, and synaptic transmission (Butterfield and Halliwell, 2019).

**FIGURE 1:**
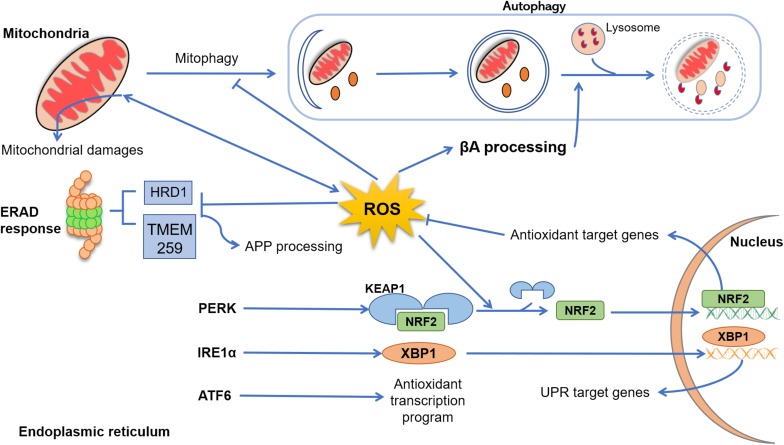
Cross talk between reactive oxygen species (ROS), endoplasmic reticulum (ER), mitochondria, and autophagy. In Alzheimer’s disease (AD), mitochondrial dysfunction progress due to aging, genetic abnormalities, environmental damage, or neuroinflammation, resulting in excessive production of ROS and accumulation of lipids’, proteins’, and nucleic acids’ damage. In physiological conditions, dysfunctional mitochondria are removed from cytoplasm through autophagy (mitophagy), an event that in AD disease is suppressed by excessive levels of ROS and β-amyloid peptide (Aβ). Emerging evidence indicated that ROS elevation may also enhance Aβ processing, inhibiting the fusion between late endosomes and lysosomes. Under several physiological and pathological conditions, the process of protein folding and post-translational modification could be overwhelmed, leading to abnormal levels of ER stress and, collectively, results in the activation of the unfolded protein response (UPR) through three sensor proteins: protein kinase RNA-like ER kinase (PERK), ATF6, and IRE (inositol-requiring protein) 1α. PERK activation leads to a conformational change that results in the release of Nrf2. This release is enhanced by ROS-induced changes, and Nrf2 translocates to the nucleus, where it induces the expression of genes coding antioxidant proteins. Upon stimulation by ER stress, IRE1α processes X-box–binding protein (Xbp1) transcripts, resulting in a splice variant of Xbp1 mRNA (Xbp1s) that encodes the transcription factor XBP1s. Finally, XBP1s translocates to the nucleus and induces an array of genes involved in the recovery of ER functions. After activation, ATF6 translocates to the Golgi, where it is cleaved by two proteases and its cytoplasmic domain is translocated to the nucleus, turning up an antioxidant program. Clearance of misfolded and damaged proteins could be also solved by ER-associated degradation. However, exacerbated ROS levels inhibit the activity of proteins such as HRD1 and TMEM259, resulting in an enhancement of amyloid precursor protein (APP) accumulation and Aβ production.

Increased OS in AD brains may also result in damage to mtDNA, lipids, and proteins (Larosa and Remacle, 2018; Figure 1). In fact, mtDNA exhibits higher levels of oxidized nucleotide bases compared to nuclear DNA, which suggests differential ROS−induced damage (Krishnan et al., 2012). Additionally, cell lines containing mtDNA from AD patients displayed an increased ROS and free radical production, compared to controls (Swerdlow et al., 1997). Besides, mutations in genes encoding mitochondrial proteins have been reported to be modified by ROS in AD. Swerdlow et al. (1997) used a cybrid model in which they replaced endogenous mtDNA from Ntera2Dl (NT2) teratocarcinoma cells with platelet mtDNA from AD patients, observing a reduced cytochrome c oxidase (COX) activity, and depletion of endogenous mtDNA changes the enzyme deficits observed.

Aβ has been proven to induce direct changes in neuron cells at the mitochondrial level. Indeed, increased levels of Aβ have been found in the mitochondria of human post-mortem AD brain as well as in transgenic mouse models overexpressing mutant amyloid precursor protein (APP) (Devi, 2006; Pavlov et al., 2009). In this regard, APP contains a mitochondrial targeting signal and an internal sequence enriched in acidic amino acids, which can form stable complexes with the translocase of the outer membrane 40 (TOM40) and the translocase of the inner mitochondrial membrane 23 (TIM23), this translocation being independent of the membrane potential, thus differing from the common mechanism of mitochondrial protein import (Hansson Petersen et al., 2008). Accumulation of APP into mitochondria membranes and cristae alters the normal function of key mitochondrial enzymes such as COX, pyruvate dehydrogenase, and α-ketoglutarate dehydrogenase complex (Casley et al., 2001). However, the mechanisms by which exogenously added Aβ peptide is trafficked to mitochondria remain yet unclear.

The electron transport chain (ETC) is also damaged in AD as well as in other neurodegenerative diseases such as Parkinson’s disease (PD) (Parker et al., 1994, 2008). In this concern, there is increasing evidence supporting the idea that impairment of the respiratory complexes in ETC is linked to both aging and AD. Rhein et al. (2009) generated triple-transgenic AD mice (cross-breeding of tau transgenic pR5 mice and APP^sw^PS2^N141I^ double-transgenic APP152 mice) and found a significative age-associated deregulation of mitochondrial proteins, mainly related to complexes I and IV of the ETC (Rhein et al., 2009). In clinical studies, COX subunits II and IV are decreased during aging but are also even more diminished in AD (Ojaimi et al., 1999). Also, platelets from AD patients have reduced COX activity, which is associated with an overproduction of ROS and an impaired ATP production (Cardoso et al., 2004). In PD, a similar reduction is observed in the activity of complex I in the *substantia nigra* of *postmortem* samples from PD patients (Gatt et al., 2016).

Aβ enhances OS (Cheignon et al., 2018). Aβ-induced OS theory hypothesized that Aβ_1__–__42_ inserted as oligomers into the lipid bilayer serve as a source of ROS, initiating lipid peroxidation, protein oxidation, and formation of ROS and RNS (Butterfield et al., 2001). One of the most established explanations of this phenomenon is based on the modulation of metal homeostasis through coordination of Aβ with metal ions Zn^2+^, Cu^2+^, and Fe^2+^. These metal ions play a significant role in both production and defense against ROS and are required to regulate the neuronal activity in the synapses and other biological functions in the brain (Cheignon et al., 2018). Notably, Cu^2+^ levels can be increased up to three times in AD patients and are usually found in the surroundings of amyloid plaques (Lovell et al., 1998). In the presence of a reducing agent, redox active metal ions such as Cu^2+^ and Fe^2+^ can have catalytic activity and form complexes with Aβ (Cheignon et al., 2018). For instance, the coordination of Cu^2+^ with Aβ also forms a stable complex that catalyzes the formation of H_2_O_2_ and ⋅OH in the presence of O_2_, and a reducing agent Fe^2+^ can be also coordinated to Aβ but has a lower redox activity than the Cu^2+^/Aβ system (Nakamura et al., 2007).

## Oxidative Stress and Proteostasis

The ER is a vital cellular organelle in eukaryotes in which occurs the synthesis and folding of the vast majority of secretory and membrane proteins (Erbaykent Tepedelen and Ballar Kirmizibayrak, 2019). To prepare the nascent proteins properly for an extra-cellular fate, the ER lumen holds a specialized environment for high-fidelity protein folding and assembly (Díaz-Villanueva et al., 2015). This efficiency is firmly anchored to the high concentrations of chaperones and folding enzymes, which allow proteins’ maturation (Adams et al., 2019). Moreover, ER also possesses oxidizing components, which favors the formation of disulfide bonds (Bulleid, 2012).

Endoplasmic reticulum is also responsible for the quality control of the proteins produced (Araki and Nagata, 2011). To maintain the balance between protein synthesis, degradation, and any additional post-translational processing, namely proteostasis, cells dispose of a complex array of sensors and transcriptional effectors to ensure the fidelity of protein folding and maturation (Balch et al., 2008). Only correctly folded proteins can exit the ER and travel toward their final destinations (Braakman and Hebert, 2013). However, if the amount of proteins to be folded exceeds the capacity of the folding machineries, unfolded proteins are accumulated within the ER lumen, inducing ER stress (ERS) (Malhotra and Kaufman, 2007). As shown in Figure 1, cells have an integrated signaling system to try to restore the normal ER function.

## Oxidative Stress and Unfolded Protein Response

Abnormal levels of misfolded proteins at the ER engage the unfolded protein response (UPR), a complex signaling system that correctly manages protein folding and initiates apoptosis or autophagy in irreversibly damaged cells (Gerakis and Hetz, 2018). ERS sensors include inositol-required enzyme 1 (IRE1, α, and β), protein kinase RNA-like ER kinase (PERK), and activating transcription factor (ATF) 6 (Gerakis and Hetz, 2018; Figure 1). In physiological conditions, the three transducers are maintained inactive by the chaperone binding immunoglobulin protein/78 kDa glucose-regulated protein (Bip/GRP78), but when ERS occurs, Bip/GRP78 is dissociated from the transducers, inducing UPR activation (Bertolotti et al., 2000).

The adaptive response induced by UPR can modulate ROS production within the ER by reducing the folding demand and upregulating the expression of antioxidant factors (Ma, 2013, 2). The control of ROS production by UPR is essentially linked to IRE1 and PERK pathways, in which ATF4 plays a key role in glutathione (GSH) synthesis and, therefore, in the maintenance of redox balance in the ER (Harding et al., 2003). In the ATF6 pathway, ATF6 translocates to the Golgi, where it is cleaved by two proteases, which releases its cytoplasmic domain to translocate to the nucleus, where it acts as a transcription factor. In addition to activating the expression of genes whose products mediate protein folding, ATF6 activates the transcription of an antioxidant program (McKimpson and Kitsis, 2017).

Another example of this cross talk could be observed in the production of UDP-N-acetylglucosamine (UDP-GlcNAc) by previous IRE1 branch activation. UDP-GlcNAc is crucial for stress-induced O-GlcNAc modifications, which favors cell viability and antioxidant defenses against ROS (Vincenz and Hartl, 2014). In addition to these pathways, the activation of the nuclear factor erythroid 2–related factor 2 (NRF2) is also involved in the antioxidant response. In basal conditions, NRF2 is inactivated by the Kelch-like ECH associated protein 1 (KEAP1), which induces its degradation (Ma, 2013). During OS, ROS induce conformational changes on KEAP1, preventing its binding to nascent NRF2 (Itoh et al., 2003). Moreover, p62 protein, whose expression is induced by ROS, also contributes to the activation of NRF2 by docking directly onto KEAP1 through a KEAP1 interacting region (KIR), thereby blocking binding between KEAP1 and NRF2 (Jain et al., 2010). In both scenarios, NRF2 migrates into the nucleus and activates transcription of antioxidant-related machinery (Itoh et al., 2003; Jain et al., 2010; Figure 1).

Besides, even though UPR activation could preferentially reduce abnormal production of ROS in ERS conditions, evidence shows that UPR pathways can, opposingly, aggravate OS. For instance, the activation of C/EBP homologous protein (CHOP) in the PERK pathway can upregulate the expression of the ER oxidoreductase 1 (Ero1), increasing peroxide production during oxidative protein folding (Marciniak, 2004). Furthermore, CHOP transcription can be induced by the ROS-induced activation of NADPH oxidase (NOX) member 2 or 4 (Li et al., 2010). The IRE1 pathway is also involved in OS-induced apoptosis by increasing thioredoxin-interacting protein (TXNIP) mRNA levels throughout the downregulation of inhibitory modulators, making cells more susceptible to OS since TXNIP inhibits the antioxidant thioredoxin (TRX) enzyme (Lerner et al., 2012).

In the context of AD, different perturbations in the secretory pathway correlate with ERS. *In vitro* exposure to Aβ products, fibrils or oligomers, drastically imbalances the ER calcium homeostasis, leading to abnormal protein folding (Alberdi et al., 2013). Moreover, Aβ oligomers can inhibit the proteasome, leading to ERS-mediated apoptosis (Kechko et al., 2019). In line with these results, ROS-induced mitochondrial dysfunction can also exacerbate ERS through elevation of levels of cytosolic free Ca^2+^ after the release of mitochondrial Ca^2+^ stores (Lim et al., 2009). The occurrence of pathological levels of ERS is well-known in the human AD brain (Scheper et al., 2007; Song et al., 2008). Several markers of ERS correlate with the progression of AD. Phosphorylated PERK, the main target of its downstream effector eIF2α, is highly concentrated in the hippocampal area, where they colocalize with phosphorylated tau proteins (Hoozemans et al., 2009). Phosphorylated IRE1 and the proapoptotic UPR transcription factor CHOP are also upregulated in the AD brain and directly correlate with the Braak stage (Duran-Aniotz et al., 2017).

## Oxidative Stress and Endoplasmic Reticulum-Associated Degradation

Another pathway to control the levels of proteins involves targeting them for degradation by the ubiquitin-proteasome system (UPS) (Stevenson et al., 2016). In the ER, this process is termed ER-associated degradation (ERAD) (Christianson and Ye, 2014). ERAD is a fine-tuned multistep system that recognizes, ubiquitinates, and retrotranslocates misfolded proteins and certain other target proteins from the ER to the cytosol for clearance by the cytosolic 26S proteasome (Christianson and Ye, 2014).

ERAD is subdivided into three pathways, depending on the localization of the misfolded lesion or degradation signal that is presented by the substrate protein, namely ERAD-L, ERAD-M, and ERAD-C (Erbaykent Tepedelen and Ballar Kirmizibayrak, 2019). ERAD-L is the degradation pathway that targets soluble proteins within the ER lumen or ER membrane–associated proteins with a misfolded domain facing the lumen (Erbaykent Tepedelen and Ballar Kirmizibayrak, 2019). Any factors involved in this branch are chaperones such as Derlins (Knop et al., 1996), Usa1 (Carroll and Hampton, 2010), or Sel1 in mammals (Christianson et al., 2008). ERAD-C targets ER membrane protein with a misfolded cytoplasmic-facing domain and used cytoplasmic chaperones and a different E3 ligase, Doa10 (Erbaykent Tepedelen and Ballar Kirmizibayrak, 2019). The ERAD-M branch targets integral membrane proteins with folding damages within the lipid bilayer (Erbaykent Tepedelen and Ballar Kirmizibayrak, 2019). Hrd1 ubiquitin ligase and the associated Hrd3 cofactor seem to be essential to ubiquinate these ERAD-M substrates (Sato et al., 2009). Despite the temporal and spatial specificity of these pathways, most misfolded integral membrane proteins will follow more than one of these three ERAD pathways (Kota et al., 2007).

Some studies indicate cross talk between ERAD pathways and OS in AD. For instance, inhibition of HRD1 expression correlates with APP accumulation and Aβ production (Yang et al., 2013). TMEM259, an ERAD component that mediates degradation of ER luminal and membrane substrates, enhances γ-secretase activity and neuronal degeneration when it is knocked out (Zhu et al., 2017). Tau accumulation also impairs ERAD by binding to HRD1, which leads to increasing misfolded protein aggregates and activation of UPR (Abisambra et al., 2013).

## Oxidative Stress and Autophagy in Alzheimer’s Disease

Autophagy is an adaptative process induced under different forms of stress, including nutrient deprivation, hypoxia, and infection (Peña-Oyarzun et al., 2018). Physiological functions of autophagy comprise elimination of macromolecules and organelles (protein and organelle turnover), cellular differentiation, programed cell death, and transportation of target proteins from the cytoplasm to the lysosomal/endosomal compartments (Mizushima, 2009). It is a complex process that consists of several sequential steps, sequestration, degradation, and amino acid/peptide generation, mediated by a unique organelle called the autophagosome, a double-membraned vesicle that contains cellular material targeted to be degraded by an intracellular degradation system (Mizushima, 2009).

Although all cell types have the ability to turn on autophagy, a growing body of studies suggest the importance of this process, specifically, in neurons (Alirezaei et al., 2008). This cell type possesses highly specialized structures and dynamic functions that require a fine-tuned intracellular control. Another feature of neurons is the postmitotic state, making them more sensitive to accumulation of toxic components than dividing cells. Therefore, quality control of neuronal components by autophagy is crucial for survival in specialized postmitotic neurons. Indeed, inhibition of autophagy events is causally linked to neurodegeneration, indicating the relevance of autophagy in the neuronal homeostasis regulation (Komatsu et al., 2006).

The selective autophagic removal of mitochondria or mitophagy is crucial in neurons, in which mitochondria play an essential role in cell survival (Lautrup et al., 2019). Neurons are particularly vulnerable to mitochondrial dysfunction due to their metabolic characteristics and postmitotic state. Thus, mitochondrial defects can significantly affect neuronal biology (Palomo and Manfredi, 2015). In fact, ROS-mediated mitochondrial injury is observed in several neurodegenerative diseases including PD (Reeve et al., 2018) or AD (Sun et al., 2008; Armand-Ugon et al., 2017), as well as in cancer (Yang et al., 2010; Zou et al., 2015), and metabolic pathologies (Liemburg-Apers et al., 2015). Both in physiological and pathological mitophagy, PTEN-induced kinase 1 (PINK1) emerged as a key regulatory pathway of mitophagy (Gkikas et al., 2018).

Other abnormalities in the autophagy pathway have been observed in several neurodegenerative diseases. Like protein homeostasis, a sustained impairment of balance between autophagosome formation and degradation causes “autophagic stress.” Either excessive autophagic demand or defects of fusion or lysosomal degradation of autophagic vacuoles (AVs) could cause autophagic stress (Chu, 2006). There is substantial evidence that deregulation of autophagy occurred in AD patients and AD animal models. Early in the 1960s, Suzuki found a large amount of hyper-phosphorylated tau protein and intracellular vesicles accumulated in neurites in AD patient brains (Suzuki and Terry, 1967), but the identity of these immature AVs was still unknown until 2005 (Nixon et al., 2005). In the same year, surprising results from AD mouse models showed AV even before Aβ plaques appeared in the mice (Yu et al., 2005). In this regard, both genetic and environmental factors connect autophagy with AD. Apolipoprotein E4 (apoE4) overexpression, the main genetic risk factor for late-onset AD, could cause a hyper-generation of Aβ42 in lysosomes, leading to neuronal death in the hippocampus (Ji et al., 2006; Belinson et al., 2008).

Conversely, the cross talk among OS and autophagy in AD remains elusive, even though previous studies highlight the relevance of this communication. For instance, SH-SY5Y neuroblastoma cells exposed to OS accumulated more Aβ-enriched lysosomes than the control group, indicating the regulation of autophagy through OS (Zheng et al., 2011). In fact, other authors have suggested an OS-induced enhancement of APP processing and a decrease in Aβ elimination because of either a downregulation process in the lysosomal degradation pathway or a structural modification of Aβ, leading to a more hydrolysis-resistant form (Muche et al., 2017).

## Therapeutic Approaches

Restoring mitochondrial function by antioxidant supplementation therapy is an unexplored but potential strategy for AD patients (Van Giau et al., 2018). One of the most common antioxidants is Ubiquinone, the oxidized form of CoQ10, which relays electrons for ATP production in the ETC and regulates OS and inflammation (Pala et al., 2018). Several studies have shown the protective action of CoQ10 against Aβ-induced neurotoxicity in endothelial and neuronal cells (Choi et al., 2013; Durán-Prado et al., 2014). However, to date, there are no published results from clinical trials of CoQ10 in AD (Young et al., 2007). Idebenone, a lipophilic synthetic analog of CoQ10, was also shown to alleviate neuroinflammation *in vitro* and *in vivo* models (Yamada et al., 2015). Nevertheless, idebenone treatment failed to improve cognitive function in AD patients (Thal et al., 2003).

About misfolding protein aggregations, various Sirtuin 1 (Sirt1) enhancers have been proven to reduce misfolding protein-induced neurotoxicity, such as resveratrol, SRT1460, and SRT1720 (Drygalski et al., 2018; Min et al., 2018). One approach using BACE1 inhibitors in AD was promising few years ago, but the increase of BACE1 expression by any BACE1 inhibitors raises concern about their use for AD (Liu et al., 2019). The compound IRL-1620, an endothelin receptor type B agonist, stimulates the clearance of Aβ and cerebral blood flow, reducing OS and improving memory impairment in rat models (Briyal et al., 2014, 2015; Pozhilenkova et al., 2017; Gulati et al., 2018). Another reduction approach for protein aggregates is based on immunotherapy by passive immunization (Salloway et al., 2014; Sevigny et al., 2016). Passive immunization of PDAPP mice (a classical mouse model of AD) with antibodies against Aβ peptide reduces plaque burden, triggering microglial cells to clear plaques through Fc receptor-mediated phagocytosis (Bard et al., 2000). Indeed, a patient immunized with AN-1792 (an adjuvanted formulation with Aβ-42 antibodies) showed decreased amyloid aggregates in the frontal cortex with no clinical evidence of inflammation (Masliah et al., 2005).

To palliate the effects of the autophagy dysregulation present in AD, various herbal compounds have been demonstrated to exert neuroprotective activity and/or improve memory deficiency in AD patients (Zeng et al., 2019). In this regard, ginsenoside-Rg2 could induce mTOR-independent autophagy, enhancing the removal of cerebral Aβ in a 5 × FAD mouse model of AD (Fan et al., 2017). Plant-derived alkaloids are also a desirable choice to increase autophagic flux and Aβ degradation in hippocampus neurons (Li et al., 2017). Additionally, there are some other compounds such as polyphenols (curcumin, resveratrol, and emodin, …) that are attracting widespread attention as modulators of autophagy (Zeng et al., 2019). *In vitro* and *in vivo* studies showed the anti-inflammatory and neuroprotective effects of resveratrol through activating mitophagy in AD (Gomes et al., 2018). Despite the promising preliminary results, only few clinical trials have been published using resveratrol in AD with satisfactory results (Berman et al., 2017).

## Conclusion

**FIGURE 2:**
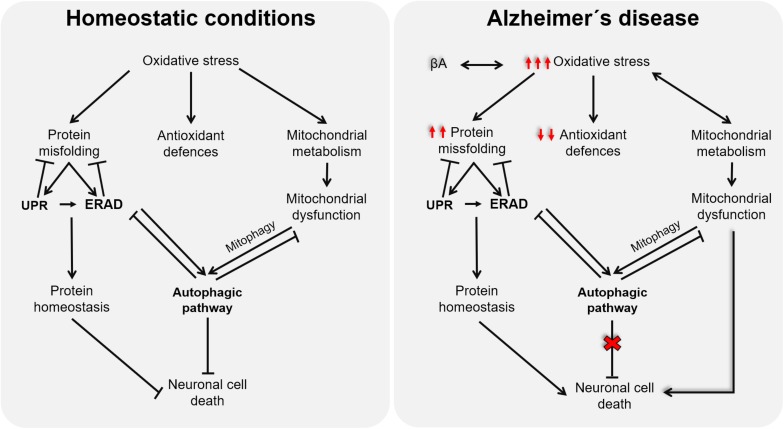
Schematic representation of the cross talk among oxidative stress (OS), endoplasmic reticulum stress, and mitochondria function.

Although the onset of AD remains elusive, multiple lines of evidence support OS as a promising candidate for the disease’s etiology. The impairment of mitochondrial activities, proteostasis network, and/or autophagic processes caused by redox imbalance in the context of AD (as illustrated in Figure 2) highlights the relevance of new antioxidative approaches, able to target all related cellular processes. However, the partial deceleration demonstrated in AD trials through antioxidative interventions could be because they are conducted as palliative and not as preventive, which is currently limited by the lack of premorbid and prodromal markers of the disease. Further studies oriented to discover these early predictive markers will surely open a new therapeutic window, before tissues are damaged, to test novel approaches for AD, in which antioxidants targeting mitochondrial dysfunction, ERS, autophagy, and ERAD will surely have a promising role.

## Author Contributions

All authors contributed to the article review, structure, and writing of the manuscript.

## Conflict of Interest

The authors declare that the research was conducted in the absence of any commercial or financial relationships that could be construed as a potential conflict of interest.
